# Effect of the GLP‐1 receptor agonist exenatide on pro‐inflammatory and metabolic biomarkers in individuals with alcohol use disorder: Post hoc results from a randomized, double‐blinded, placebo‐controlled clinical trial

**DOI:** 10.1111/acer.70110

**Published:** 2025-07-09

**Authors:** Malthe E. B. Hviid, Lea A. N. Christoffersen, Mette K. Klausen, Thorsten Brodersen, Ole B. Pedersen, Sisse R. Ostrowski, Margit H. Larsen, Mette Kongstad, Mathias E. Jensen, Tina Vilsbøll, Anders Fink‐Jensen

**Affiliations:** ^1^ Psychiatric Centre Copenhagen, Mental Health Services in the Capitol Region of Denmark Copenhagen Denmark; ^2^ Department of Clinical Immunology Zealand University Hospital Koege Denmark; ^3^ Institute of Biological Psychiatry, Mental Health Services Copenhagen Copenhagen University Hospital Copenhagen Denmark; ^4^ Department of Clinical Medicine, Faculty of Health and Medical Sciences University of Copenhagen Copenhagen Denmark; ^5^ Department of Clinical Immunology University of Copenhagen, Rigshospitalet Copenhagen Denmark; ^6^ Steno Diabetes Center Copenhagen University of Copenhagen Copenhagen Denmark

**Keywords:** alcohol use disorder, exenatide, FGF‐21GIP, GLP‐1, hsCRP, IL‐6

## Abstract

**Background:**

Alcohol use disorder (AUD) has been associated with inflammation, metabolic syndrome, and increased risk of all‐cause mortality. This study aimed to compare the pro‐inflammatory and metabolic biomarker profiles in individuals with AUD with individuals without AUD, and to evaluate the effect of exenatide on these biomarkers in individuals with AUD.

**Methods:**

Serum concentrations of 25 biomarkers (interferon‐γ [IFN‐γ], tumor necrosis factor‐α [TNF‐α], interleukin (IL)‐1β, IL‐2, IL‐4, IL‐6, IL‐8, IL‐10, IL‐12p70, IL‐13, monocyte chemoattractant protein‐1 [MCP‐1], C‐peptide, gastric inhibitory polypeptide [GIP], glucagon‐like peptide [GLP‐1], glucagon, insulin, leptin, pancreatic polypeptide [PP], adiponectin, high sensitivity C‐reactive protein [hsCRP], fibroblast growth factor 21 [FGF‐21], total cholesterol [CHOL], high‐density lipoprotein [HDL], low‐density lipoprotein [LDL], and triglycerides [TG]) from individuals with AUD were measured at baseline and after 26 weeks of treatment with the GLP‐1 receptor agonist (GLP‐1RA) exenatide once‐weekly or placebo, using multiplexed immunoassays, enzyme‐linked immunosorbent assay (ELISA), and line immunoassays. Serum samples from 23 individuals with no record of AUD or treatment with a GLP‐1RA were measured once for comparison with individuals with AUD.

**Results:**

IL‐6 (1.56 vs. 0.62 pg/mL), hsCRP (3.30 vs. 1.34 mg/L), and FGF‐21 (1794.97 vs. 306.11 pg/mL) were significantly higher, whereas GIP (63.06 vs. 111.07 pg/mL) was significantly lower in individuals with AUD (*n* = 124) than in those without AUD (*n* = 23). No significant changes in biomarker levels were observed after treatment with exenatide (*n* = 40) compared with treatment with placebo (*n* = 37).

**Conclusion:**

Our findings support the well‐established link between AUD and inflammation. However, treatment with the GLP‐1 receptor agonist exenatide did not impact pro‐inflammatory and metabolic biomarkers.

## Graphical abstract

This study compared pro‐inflammatory and metabolic biomarker profiles between individuals with alcohol use disorder (AUD) and those without, and evaluated the impact of the GLP‐1 receptor agonist exenatide on these biomarkers in the AUD group. At baseline, individuals with AUD exhibited significantly elevated levels of IL‐6, hsCRP, and FGF‐21, and reduced GIP levels compared to controls, underscoring the link between AUD and systemic inflammation. However, treatment with exenatide did not result in significant changes in biomarker levels compared to placebo.

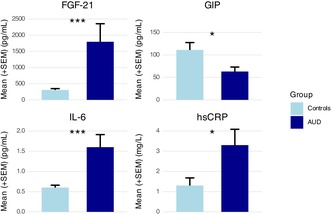

## INTRODUCTION

Alcohol use disorder (AUD) affects approximately 5% of the global population and is associated with high morbidity and mortality (Askgaard et al., 2019; Carvalho et al., 2019).

Use of alcohol and drugs has been shown to activate the same reward pathway as food reward (Volkow et al., 2013), and glucagon‐like peptide‐1 receptor agonists (GLP‐1RAs) have been found in areas associated with reward and addiction in rodents and nonhuman primates (Fink‐Jensen et al., 2025; Heppner et al., 2015; Jensen et al., 2017). Additionally, GLP‐1RAs significantly reduce alcohol consumption and alcohol‐related behavior in nonhuman primates and rodents (Brunchmann et al., 2019; Fink‐Jensen et al., 2025; Klausen, Thomsen, et al., 2022; Thomsen et al., 2019). However, we were unable to detect a similar effect in a double‐blinded, randomized, placebo‐controlled clinical trial conducted among 127 individuals with AUD, evaluating the efficacy of the GLP‐1RA exenatide at a dose of 2 mg administered once‐weekly, over the course of 26 weeks (Klausen, Jensen, et al., 2022).

In human studies, higher blood levels of multiple pro‐inflammatory cytokines have been found in individuals with AUD, such as tumor necrosis factor‐α TNF‐α, which is associated with AUD severity (Heberlein et al., 2014), and interleukin (IL)‐1β, IL‐6, and IL‐8, which are associated with alcohol craving (Heberlein et al., 2014; Leclercq et al., 2014). A systematic review and meta‐analysis comparing individuals with and without AUD found that IL‐6 was significantly elevated in individuals with AUD (Moura et al., 2022).

In addition to an inflammatory response, individuals with AUD have a twofold risk of cardiovascular disease (CVD) (Kozela et al., 2020; Sung et al., 2022) and cardiovascular mortality compared with the general population (Roerecke & Rehm, 2014). Several clinical studies have demonstrated that treatment with GLP‐1RAs can effectively prevent cardiovascular events such as myocardial infarction and stroke in individuals with overweight or obesity and type 2 diabetes (Lincoff et al., 2023; Sattar et al., 2021). It has been proposed that the anti‐inflammatory effects of GLP‐1RAs coupled with its lowering effects on systolic blood pressure and body weight, along with reducing plasma low‐density lipoprotein (LDL) cholesterol and triglyceride concentrations, are likely what exert its cardioprotective and atheroprotective effects (Chaudhuri et al., 2012; Nauck et al., 2021).

In this study, we investigate the pro‐inflammatory and metabolic biomarker profiles in the serum of individuals with AUD compared to those without AUD. Additionally, we evaluate the potential effects of the GLP‐1RA exenatide on pro‐inflammatory and metabolic biomarker levels in individuals with AUD. We hypothesize that concentrations of pro‐inflammatory cytokines and metabolic biomarkers are increased in individuals with AUD compared to individuals without AUD and that treatment with the GLP‐1RA exenatide will normalize levels of pro‐inflammatory and cardiometabolic biomarkers in individuals with AUD.

## METHODS

### Study design and sample

**FIGURE 1:**
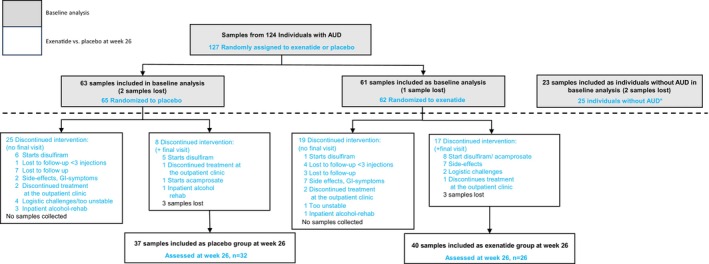
Flowchart of sample collection and analysis. AUD, alcohol use disorder. *25 individuals without alcohol use disorder included as a control group for the MRI brain scans.

The serum samples used in this study were derived from a randomized, placebo‐controlled, double‐blinded clinical trial conducted at outpatient alcohol clinics in Copenhagen, Denmark (Klausen, Jensen, et al., 2022). Participants were diagnosed with AUD and were aged 18–70 years. Key exclusion criteria were severe psychiatric or somatic comorbidity and concomitant pharmacotherapy against AUD. A total of 127 individuals were recruited for the study (*n* = 65 in the placebo group and *n* = 62 in the exenatide group), of which 58 participated in a Week 26 follow‐up (*n* = 32 in the placebo group and *n* = 26 in the exenatide group). Reasons for trial discontinuations were diverse (Figure 1). The design and results of the trial have been described in detail previously (Antonsen et al., 2018; Klausen, Jensen, et al., 2022). Individuals without AUD (*n* = 25) were included as a control group for the functional magnetic resonance imaging (fMRI) brain scans, and matched on sex, age, and educational status. The comparison group had a very low mean Alcohol Use Disorders Identification Test (AUDIT) score of 3.3, while the corresponding scores in the placebo and exenatide groups were 25.9 and 25.6, respectively (Klausen, Jensen, et al., 2022).

### Outcome measures

The following 25 outcomes were measured in this study: Interferon‐γ (IFN‐γ), IL‐1β, IL‐2, IL‐4, IL‐6, IL‐8, IL‐10, IL‐12p70, IL‐13, TNF‐α, Insulin, C‐peptide, glucagon‐like peptide 1 (GLP‐1), gastric inhibitory polypeptide (GIP), glucagon, pancreatic peptide (PP), leptin, fibroblast growth factor 21 (FGF‐21), monocyte chemoattractant protein‐1 (MCP‐1), adiponectin, high sensitivity C‐reactive protein (hsCRP), total cholesterol, LDL, high‐density lipoprotein (HDL), and triglycerides.

### Blood sample analysis

Serum samples (*n* = 234) were analyzed using multiplexed immunoassays (Meso Scale Discovery©): V‐Plex Pro‐inflammatory Panel 1 (human), V‐Plex Metabolic Panel 1 (human), and a customized U‐PLEX panel for FGF‐21 and MCP‐1 on the Meso QuickPlex 120 (MSD) and the Quantikine™ ELISA (R&D Systems®) for Adiponectin. Additionally, line immunoassays (LIA) of hsCRP, total cholesterol, LDL, HDL, and triglycerides were performed on a Cobas 8000 (Roche Diagnostics). The V‐PLEX assays are validated according to fit‐for‐purpose principles (Lee et al., 2006) and design control procedures of the providing company, Meso Scale Discovery. Further details, including assay sensitivity, precision, and stability, are found in the assay protocols (Meso Scale Discovery). All experimental procedures were performed according to the protocol provided by the manufacturer (Meso Scale Discovery).

### Blood sampling

The serum samples were collected at variable time points during the day without an overnight fast. Full blood was collected in Vacuette CAT serum clot activator tubes (9 mL) and stored at room temperature for 1–2 h before 15 min of centrifugation at 4°C (39.2°F) and 3819 g (1.100 rcf). All serum samples were immediately stored at −20°C (− 4°F) and, within 2 months, transferred to −80°C (−112°F).

### Data processing

Raw data from the Mesoscale assays were processed using the MSD DISCOVERY WORKBENCH® version 4.0 analysis software (Meso Scale Discovery, 2013). The software calculated the concentrations of analytes from the 8‐point standard linear regression made for each plate, subtracting the background value (Møller et al., 2022). Subsequently, the results were merged in an R script, and concentrations of samples below the lower limit of detection were imputed from a uniform distribution between zero and the lower limit of detection to reflect physiological concentrations more accurately than without measurement (Kwak & Kim, 2017). Concentrations of samples above the upper limit of detection were substituted with the value of the upper limit of detection.

### Statistical analyses

Baseline differences between the placebo and exenatide group, as well as between the study sample of individuals with AUD and individuals without AUD, were displayed by means with standard deviations (SD). Differences were tested with a *t*‐test and for this particular analysis, some outcome measures were log‐transformed to accommodate the assumption of a normal distribution (Table 1).

**TABLE 1:** Demographic characteristics of individuals with AUD compared with individuals without AUD and among individuals randomized to exenatide and placebo.

	Study sample of individuals with AUD and individuals without AUD		Study sample comprising individuals with AUD randomized to placebo or exenatide	
	Individuals with AUD (*n* = 124)	Individuals without AUD (*n* = 23)	Placebo (*n* = 63)	Exenatide (*n* = 61)
Age, mean (SD)	52.1 (10.3)	49.1 (8.9)	52.2 (9.9)	52.0 (10.8)
Sex, *n* (%)				
Female	50 (40.3)	10 (43.5)	26 (41.3)	24 (39.3)
Male	74 (59.7)	13 (56.5)	37 (58.7)	37 (60.7)
BMI, *n* (%)				
<25.0	50 (40.3)	7 (30.4)	22 (34.9)	28 (45.9)
25.0–29.9	44 (35.5)	12 (52.2)	26 (41.3)	18 (29.5)
30.0–34.9	22 (17.7)	3 (13.0)	12 (19.1)	10 (16.4)
≥35	8 (6.5)	1 (4.4)	3 (4.8)	5 (8.2)

Abbreviations: AUD, alcohol use disorder; BMI, body mass index.

Missing data were handled by multiple imputations. In total, 100 rounds of imputations were performed, resulting in 100 complete datasets for all outcome measures. Each dataset was imputed using the fully conditional specification (imputation by chained equations) and the predictive mean matching method. All imputation models included the variable BMI and selected auxiliary variables that were observed to correlate >0.4 with the outcome measure under imputation (Table 2). The statistical analyses for the outcome measures described below were conducted in each of the 100 datasets, and the parameter estimates were pooled to obtain the final parameter estimates.

**TABLE 2:** Biomarker concentrations at baseline in individuals with AUD compared with individuals without AUD and in individuals randomized to exenatide and placebo, respectively.

	Comparison of individuals with AUD and individuals without AUD^a^		Comparison of individuals with AUD, treated with placebo or exenatide^a^	
	Individuals with AUD (*n* = 124)	Individuals without AUD (*n* = 23)	Placebo (*n* = 63)	Exenatide (*n* = 61)
	Mean (SD)	Mean (SD)	Mean (SD)	Mean (SD)
C‐peptide, pg/mL	2026.9 (1480.3)^b^	2275.2 (1389.8)^b^	1681.9 (957.9)^b^	2383.3 (1813.7)^b^
FGF‐21, pg/mL	1795.0 (3060.9)^b^ ^,^***	306.1 (214.8)^b^	2151.2 (3751.8)^b^	1427.1 (2095.4)^b^
GIP, pg/mL	63.1 (54.9)^b^ ^,^**	111.1 (82.3)^b^	48.4 (39.3)^b^ ^,^*	78.2 (64.2)^b^ ^,^*
GLP‐1, pM	12.4 (5.2)^b^	15.8 (10.6)^b^	11.8 (4.9)^b^	13.1 (5.5)^b^
Glucagon, pM	7.8 (4.2)^b^	11.4 (11.3)^b^	7.5 (3.7)^c^	8.2 (4.7)^c^
IFN‐γ, pg/mL	8.0 (8.9)^c^	5.9 (4.4)^c^	7.5 (5.9)^c^	8.5 (11.3)^c^
IL‐10, pg/mL	0.3 (0.4)^b^	0.4 (0.3)^b^	0.3 (0.2)^b^	0.3 (0.5)^b^
IL‐12p70, pg/mL	0.4 (0.4)^b^	0.4 (0.4)^b^	0.4 (0.3)^b^	0.4 (0.4)^b^
IL‐13, pg/mL	1.0 (0.8)^b^	1.2 (1.4)^b^	1.1 (0.9)^c^	0.8 (0.6)^c^
IL‐1β, pg/mL	0.2 (0.7)^d^	0.2 (0.3)^d^	0.2 (0.7)^d^	0.2 (0.6)^d^
IL‐2, pg/mL	0.5 (1.0)^b^	0.3 (0.3)^b^	0.6 (1.3)^b^	0.4 (0.5)^b^
IL‐4, pg/mL	0.1 (0.2)^d^	0.1 (0.1)^d^	0.1 (0.1)^d^	0.1 (0.2)^d^
IL‐6, pg/mL	1.6 (1.7)^b^ ^,^***	0.6 (0.3)^b^	1.6 (1.9)^b^	1.5 (1.4)^b^
IL‐8, pg/mL	17.2 (16.0)^b^	12.7 (6.1)^b^	19.1 (20.5)^b^	15.3 (9.0)^b^
Insulin, *μ*U/mL	12.8 (13.4)^b^	14.4 (14.2)^b^	10.3 (10.2)^b^	15.5 (15.8)^b^
Leptin, pg/mL	5985.4 (6348.6)^b^	3877.9 (4842.2)^b^	6068.9 (6320.3)^b^	5899.2 (6429.1)^b^
MCP‐1, pg/mL	328.6 (119.7)	322.1 (86.5)	333.3 (123.0)	323.8 (117.0)
PP, pg/mL	216.3 (194.7)^b^	210.8 (202.2)^b^	200.4 (184.7)^b^	232.7 (204.7)^b^
TNF‐α, pg/mL	1.3 (1.0)^d^	1.1 (0.4)^d^	1.2 (0.4)^d^	1.4 (1.3)^d^
Adiponectin, ng/mL	10,380.5 (5466.5)^b^	8008.1 (4336.8)^b^	11,025.6 (4986.2)^b^	9714.3 (5889.0)^b^
CHOL, mmol/L	6.4 (1.5)	5.6 (1.0)	6.4 (1.5)	6.4 (1.4)
HDL, mmol/L	1.9 (0.8)^b^	1.6 (0.5)^b^	2.0 (0.8)^b^	1.8 (0.7)^b^
hsCRP, mg/L	3.3 (4.3)^b^ ^,^*	1.3 (1.9)^b^	3.6 (5.0)^b^	3.0 (3.4)^b^
LDL, mmol/L	3.5 (1.2)	3.2 (0.7)	3.5 (1.2)	3.5 (1.2)
TG, mmol/L	1.9 (1.1)^b^	1.7 (0.7)^b^	1.7 (0.9)^b^	2.0 (1.2)^b^

Abbreviations: AUD, alcohol use disorder; CHOL, total cholesterol; FGF‐21, fibroblast growth factor 21; GIP, gastric inhibitory polypeptide; HDL, high‐density lipoprotein cholesterol; hsCRP, high sensitivity C‐reactive protein; IFN‐γ, interferon‐γ; IL, interleukin; LDL, low‐density lipoprotein cholesterol; MCP‐1, monocyte chemoattractant protein‐1; PP, pancreatic polypeptide; SD, standard deviation; TG, triglycerides; TNF‐α, tumor necrosis factor‐α.

^a^ Comparison by Bonferroni corrected *t*‐test.

^b^ Log transformed for *t*‐test.

^c^ Log transformation of 1 + variable for *t*‐test.

^d^ Log_10_ transformed for *t*‐test.

*p ≤ 0.05; **p < 0.01; ***p < 0.001.

The change in each outcome measure was compared between the placebo and exenatide groups in generalized linear mixed models. Hence, a total of 25 models were constructed, using an indicator variable, describing whether an individual belonged to the placebo or exenatide group. The indicator variable entered the model as a fixed effect. For each model, four different types of variance–covariance structures (compound symmetry, unstructured, autoregressive, and autoregressive heterogeneous) were tested to assess what underlying structure fitted the model best. The variance–covariance structure with the lowest −2 log likelihood scores and Akaike information criterion (AIC) value was chosen for each model (Table 2).

The assumptions of the generalized linear mixed models were assessed graphically, and no deviations were observed. All analyses were performed using SAS Enterprise Guide 8.3, with a significance level of 0.05 after applying the Bonferroni correction for multiple tests.

### Ethical considerations

The study was approved by the Danish Ethics Committee of the Capital Region, Copenhagen, Denmark (H‐17003043), the Danish Data Protection Agency (RHP‐2017–029), the Danish Medical Agency (EudraCT 2016–003343–11), and ClinicalTrials.gov identifier NCT03232112.

## RESULTS

### Study sample

A total of 124 individuals with AUD had baseline serum samples analyzed (*n* = 63 from the placebo group and *n* = 61 from the exenatide group). A total of 77 samples were available for follow‐up analyses, originating from participants who finished the study at 26 weeks, or discontinued the study after ≥8 weeks but had their final serum sample collected (*n* = 37 from the placebo group and *n* = 40 from the exenatide group). At baseline, 23 samples from individuals without AUD were analyzed (Figure 1).

### Baseline comparison of individuals with and without AUD

At baseline, the serum concentrations of FGF‐21 (mean difference [MD]: 1488.86 pg/mL; *p* < 0.001), IL‐6 (MD: 0.94 pg/mL; *p* < 0.001), and hsCRP (MD: 1.96 mg/L; *p* < 0.05) were significantly higher, and GIP‐total (MD: 48.01 pg/mL; *p* < 0.01) significantly lower in the AUD group than in individuals without AUD (Table 2).

### Follow‐up comparison of the exenatide group and the placebo group

At 26 weeks of follow‐up, no significant differences were observed in any of the 25 biomarkers after Bonferroni corrections (Table 3).

**TABLE 3:** Estimated treatment effect of exenatide compared with placebo in individuals with AUD.

Biological marker	Placebo		Exenatide		Estimated treatment effect	Bonferroni corrected *p*‐value
	Baseline	Follow‐up	Baseline	Follow‐up		
	Mean		Mean		*B* (95% CI)^a^	
C‐peptide, pg/mL^b^	1681.9	2216.4	2383.3	2193.8	301.6 (−132.4; 735.6)	NS
FGF‐21, pg/mL^b^	2151.2	2082.6	1427.1	1555.0	−11,050.0 (−23,068.1; 968.5)	NS
GIP, pg/mL^b^	48.4	82.7	78.2	81.0	178.4 (20.2; 336.6)*	NS
GLP‐1, pM^b^	11.8	14.3	13.1	14.5	10.4 (−6.2; 27.0)	NS
Glucagon, pM^b^	7.5	9.0	8.2	8.0	0.2 (−1.2; 1.6)	NS
IFN‐γ, pg/mL^b^	7.5	13.1	8.5	10.0	0.7 (−2.4; 3.8)	NS
IL‐10, pg/mL^b^	0.3	0.3	0.3	0.4	0.1 (−0.1; 0.2)	NS
IL‐12p70, pg/mL^b^	0.4	0.4	0.4	0.4	0.0 (−0.1; 0.1)	NS
IL‐13, pg/mL^b^	1.1	1.0	0.8	0.8	−0.3 (−0.5; 0.0)*	NS
IL‐1β, pg/mL^b^	0.2	0.2	0.2	0.1	−0.1 (−0.2; 0.1)	NS
IL‐2, pg/mL^b^	0.6	0.5	0.4	0.4	−0.2 (−0.5; 0.1)	NS
IL‐4, pg/mL^b^	0.1	0.1	0.1	0.1	0.0 (0.0; 0.0)	NS
IL‐6, pg/mL^b^	1.6	1.7	1.5	1.4	−0.2 (−0.7; 0.2)	NS
IL‐8, pg/mL^b^	19.1	22.1	15.3	17.2	−4.0 (−9.2; 1.2)	NS
Insulin, *μ*U/mL^b^	10.3	14.6	15.5	13.2	4.0 (−0.6; 8.7)	NS
Leptin, pg/mL^b^	6068.9	6148.6	5899.2	5359.3	−269.1 (−3237.5; 2699.2)	NS
MCP‐1, pg/mL^b^	333.3	338.4	323.8	337.6	−43.9 (−377.7; 289.8)	NS
PP, pg/mL^b^	200.4	255.5	232.7	291.5	57.5 (−28.6; 143.7)	NS
TNF‐α, pg/mL^b^	1.2	1.3	1.4	1.3	0.05 (−0.1; 0.3)	NS
Adiponectin, ng/mL^c^	11,026.0	9883.1	9714.3	9188.1	−1002.2 (−2805.2; 800.8)	NS
CHOL, mmol/L^c^	6.4	6.4	6.4	6.4	0.0 (−0.4; 0.4)	NS
HDL, mmol/L^b^	2.0	1.8	1.8	1.8	−0.1 (−0.3; 0.2)	NS
hsCRP, mg/L^b^	3.6	2.9	3.0	3.0	−0.1 (−1.4; 1.2)	NS
LDL, mmol/L^c^	3.5	3.5	3.5	3.5	0.1 (−0.3; 0.4)	NS
TG, mmol/L^c^	1.7	2.0	2.0	2.0	0.1 (−0.2; 0.5)	NS

Abbreviations: 95% CI, 95% confidence interval; *B*, effect estimate; CHOL, total cholesterol; FGF‐21, fibroblast growth factor 21; GIP, gastric inhibitory polypeptide; HDL, high‐density lipoprotein cholesterol; hsCRP, high sensitivity C‐reactive protein; IFN‐γ, interferon‐γ; IL, interleukin; LDL, low‐density lipoprotein cholesterol; MCP‐1, monocyte chemoattractant protein‐1; NS, not significant; PP, pancreatic polypeptide; SD, standard deviation; TG, triglycerides; TNF‐α, tumor necrosis factor‐α.

^a^ With the placebo group as the reference.

^b^ Unstructured variance–covariance structure.

^c^ Compound symmetry variance–covariance structure.

* *p* ≤ 0.05 before Bonferroni correction.

## DISCUSSION

In this double‐blinded randomized placebo‐controlled trial, the baseline concentrations of IL‐6 and hsCRP were more than doubled, FGF‐21 was increased more than sixfold, and GIP was almost halved in individuals with AUD compared with individuals without AUD. We did not observe an effect of exenatide on any of the 25 pro‐inflammatory and cardiometabolic biomarkers when compared with placebo treatment.

Our findings align with existing studies, which have shown elevated levels of pro‐inflammatory cytokines, such as IL‐6, in AUD (Adams et al., 2020; Moura et al., 2022). Both IL‐6 and hsCRP are commonly used as biomarkers of metabolic syndrome (Cho & Lee, 2022; Srikanthan et al., 2016). IL‐6 has been implicated in endothelial cell damage, vascular dysfunction, atherosclerosis, and insulin resistance through various cell signaling pathways. Increased levels of IL‐6 are associated with a more severe metabolic syndrome, as assessed by hypertriglyceridemia, hypertension, and elevated fasting glucose levels (Srikanthan et al., 2016). Elevated CRP levels are associated with metabolic disease, including dyslipidemia, diabetes, and metabolic syndrome (Cho & Lee, 2022), and are used to assess the risk of cardiovascular diseases at large (Han et al., 2022). Our findings of increased levels of IL‐6 and hsCRP in individuals with AUD compared with individuals without AUD support the link between AUD and an increased risk of metabolic syndrome or CVD reported in previous studies.

We found a sixfold increase in FGF‐21 in the AUD group compared with individuals without AUD at baseline. In humans, higher alcohol intake leads to increased levels of circulating FGF‐21, even among individuals with AUD, suggesting it to be a sensitive indicator of alcohol consumption (Farokhnia et al., 2023).

In the present study, GIP was significantly lower in individuals with AUD compared to individuals without AUD at baseline. This result is incongruent with previous reports from two smaller studies in individuals with AUD compared to individuals without AUD, finding similar levels of GIP in both groups (Fink et al., 1983; Tyler et al., 2025).

We did not observe any anti‐inflammatory or immune‐modulating effects of treatment with GLP‐1RA exenatide, which is in line with some existing studies (Courrèges et al., 2008; Møller et al., 2022) and aligns with other findings based on the same data source, showing no effect of exenatide on alcohol intake. However, other studies did report an effect of this treatment, observing reduced plasma concentration of IL‐1β, IL‐6, TNF‐α, and chemokine MCP‐1 (Bułdak et al., 2016; Chaudhuri et al., 2012).

### Strengths and limitations

This study carries several strengths that deserve mentioning. First, mesoscale multiplex assays are a well‐tested and high‐quality method of testing multiple analytes simultaneously. Second, samples from individuals allocated to the exenatide or placebo and individuals without AUD were distributed across the three plates to minimize potential effects of interassay variation. Third, the study employed a double‐blinded, randomized, placebo‐controlled trial design, and missing data were handled through multiple imputations, thereby reducing the risk of confounding and selection bias due to loss to follow‐up.

However, some potential measurement artifacts cannot be ruled out: Blood clotting of serum samples before centrifugation required 1–2 h at room temperature, potentially affecting cytokine levels (Adams et al., 2020). Furthermore, daily variation in the time of sample collection could also affect cytokine levels (Adams et al., 2020).

A relatively high proportion of missing data was observed for the outcome measures, which may have affected the risk estimates. Hence, only 26 of the 62 individuals with AUD treated with exenatide completed the entire 26 weeks of treatment. While the time‐to‐treatment discontinuation was not significantly different between the exenatide and placebo groups, the discontinuation may have impacted the statistical power to observe an effect of exenatide treatment on pro‐inflammatory and cardiometabolic biomarkers. Moreover, exenatide may not have been sufficiently potent to detect an effect on the included biomarkers. Finally, individuals with severe psychiatric disease, diabetes, and impaired renal and hepatic function were excluded, potentially affecting the generalizability of the results.

## CONCLUSION

Baseline comparisons between individuals with and without AUD revealed increased levels of IL‐6, hsCRP, and FGF‐21, and reduced levels of GIP among individuals with AUD, supporting the well‐established link between AUD and inflammation. Treatment with the GLP‐1RA exenatide did not impact pro‐inflammatory and cardiometabolic biomarkers compared with placebo treatment.

## CONFLICT OF INTEREST STATEMENT

TV has been part of speaker's bureaus, served on scientific advisory panels, served as a consultant to and/or received research support from Amgen, Boehringer Ingelheim, Eli Lilly, Gilead, AstraZeneca, Mundipharma, MSD/Merck, Novo Nordisk, and Sun Pharmaceuticals. AF‐J has received an unrestricted research grant from Novo Nordisk to investigate the effects of GLP‐1 receptor stimulation on metabolic disturbances in antipsychotic‐treated patients with a diagnosis of schizophrenia and serves on an advisory panel for Novo Nordisk (no honorarium).

## Data Availability

The data that support the findings of this study are available on request from the corresponding author. The data are not publicly available due to privacy or ethical restrictions.
